# Intestinal parasitosis and associated factors among diabetic patients attending Arba Minch Hospital, Southern Ethiopia

**DOI:** 10.1186/s13104-018-3791-x

**Published:** 2018-10-01

**Authors:** Getaneh Alemu, Abdu Jemal, Zerihun Zerdo

**Affiliations:** 1 Department of Medical Laboratory Sciences, Bahir Dar University, Bahir Dar, Ethiopia; 2 Department of Medical Laboratory Sciences, Arba Minch University, Arba Minch, Ethiopia

**Keywords:** Intestinal parasites, Diabetes mellitus, Opportunistic parasites

## Abstract

**Objective:**

Local assessment of the magnitude of intestinal parasitic infections and associated factors among diabetic patients helps for good prognosis of diabetes. Hospital based cross-sectional study was conducted by recruiting 215 diabetic patients. A structured questionnaire was used to capture data about socio-demographic characteristics, clinical history and factors associated with intestinal parasitosis. Stool samples were collected and processed by direct wet mount, formol-ether concentration and modified ziehl-Neelson staining techniques. All data were analyzed using Statistical Package for Social Sciences software version 20.

**Results:**

The rate of intestinal parasitic infection among diabetic patients was 19.5%. *Cryptosporidium parvum* accounts the highest frequency (18, 8.4%) followed by *Ascaris lumbricoides* (8, 3.7%). Presence of domestic animals in the house (AOR = 2.857, 95% CI 1.290–6.330, p = 0.010), manifestation of abdominal pain (AOR = 3.716, 95% CI 1.632–8.459, p = 0.002) and farmer and labor occupation (AOR = 3.695, 95% CI 1.082–12.618, p = 0.037) were significantly associated with intestinal parasitosis. The magnitude of intestinal parasitosis among diabetic patients attending Arba Minch Hospital was considerable. Hence, we recommend routine screening and prompt treatment for intestinal parasitosis in order to improve the health of diabetic patients.

## Introduction

Diabetes mellitus (DM) is a clinical syndrome manifested by chronic hyperglycemia. It is caused by defects in insulin secretion, action or both [1]. Diabetes mellitus results from either autoimmune destruction of insulin producing beta cells of the pancreas or insulin resistance, a condition in which cells fail to properly use insulin. Globally, an estimated 422 million adults were living with diabetes in 2014 [2]. In 2015, diabetes was the direct cause of 1.6 million deaths [3]. In sub-Saharan Africa, over 12 million people are expected to have DM, and 330,000 of these people will die from DM related complications [4]. In Ethiopia, according to the World Health Organization (WHO) estimation, the number of diabetic cases in the year 2000 was 800,000 and this number is expected to increase to 1.8 million by the year 2030 [5].

Recently, it has been demonstrated that both innate and adaptive immune responses are impaired in DM patients [6]. As a result, they are easily affected by infectious causes of morbidity. Intestinal protozoa and helminths use this opportunity to cause more aggressive and chronic form of morbidity among DM patients. Previous surveys from Iran (24.4–26.3%), Tehran province (15.6%), Cameroon (10%), Nigeria (18.7%) and Egypt (25–39%) indicated that considerable proportions of DM patients host intestinal parasites [6–12].

Intestinal parasites (IPs) are among the most widespread causes of human infections worldwide [10]. However, the species distribution differs region to region because of variations in environmental, social, and geographical factors. They spread mostly in areas with poor sanitation and are most common in tropical countries [13]. Moreover, the clinical significance and associated risk factors is more related to, and extensively studied in children considering them as at higher risk than the other population groups. However, as observed in our previous study among tuberculosis patients, adolescents and adults with certain co-existing chronic diseases are also at high risk of infection with intestinal parasites [14]. Therefore, local data about the magnitude and predominating species of parasites helps to formulate effective control strategies in order to improve the health of chronically ill people like DM patients. Despite this, there is paucity of information in Ethiopia. Hence the aim of the present study was to assess the burden of intestinal parasitosis and pre-disposing factors among DM patients attending Arba Minch Hospital, Southern Ethiopia.

## Main text

### Materials and methods

#### Study design and area

A hospital based cross-sectional study was conducted in Arba Minch diabetes clinic from March to May, 2017. Arba Minch is located in the southern part of the country about 454 kms from Addis Ababa, the capital of Ethiopia. In the town, there is one hospital (Arba Minch Hospital) and two health centers. The Hospital was built in 1961 to serve around 500,000 people, but now it is serving for more than two million people. Over 100,000 patients visit the hospital every year. The hospital gives services under different departments, of which, diabetes unit is the one. Diagnosis, treatment, counseling and follow up of DM patients are the main activities of the diabetes unit.

#### Study participants

The source population of the present study was all patients visiting diabetes clinic of Arba Minch Hospital (AMH). The study population comprises of all diabetic patients attending diabetes clinic of AMH during the study period. Those diabetic patients whose age ≥ 12 years old were included in the study but those who were seriously sick and unable to respond to the research questions and/or taking anti-parasitic drugs within 15 days before data collection were excluded. Sample size was determined using sample size determination for estimation of single population proportion formula. Taking 18.7% prevalence of IPs from previous similar study [10], 95% confidence interval (Z = 1.96) and 5% margin of error (d = 0.05), the initial sample size was 234. By considering a 5% (12 subjects) non response rate, the final sample size was determined to be 246. The sampling technique was based on systematic random selection method by calculating kth value. The number of diabetic patients visiting AMH during the same period in the year 2016 was 684. Hence K = 684/246 = 3. The first participant was recruited by lottery method and then every 3rd subject was included in the study.

#### Data collection

Data related to socio-demographic characteristics of study participants, and potential factors for intestinal parasitosis were collected by face to face interview using a pre-tested structured questionnaire. After obtaining consent from the participants, about 2 g of fresh fecal samples were collected from each participant and placed in labeled dry, clean, leak proof, tight lid plastic stool container. A portion of stool was examined in Arba Minch Hospital Parasitology laboratory by direct wet mount with saline (0.85% sodium chloride solution) to detect the presence of motile parasites and trophozoites within 30 min after collection. The remaining samples were preserved using 10% v/v formol saline and transported to Microbiology and Parasitology Laboratory of Arba Minch University for further process. A portion of the preserved stool was processed by the formol-ether concentration technique for microscopic detection of ova and cyst of IPs. The modified ziehl-Neelson staining technique was used to detect oocysts of intestinal coccidia like *cryptosporidium, Isospora,* and *cyclospora* spp. All laboratory procedures were conducted following standard protocols [15].

#### Statistical analysis

Data were cleaned, entered and analyzed using Statistical Package for Social Sciences software version 20. Descriptive statistics were calculated to describe the study population characteristics. Bivariate regression was used to assess associations between dependent and independent categorical variables. Multivariate regression model then followed for variables with p ≤ 0.25 in the bivariate analysis. Association between variables was considered statistically significant only if p-value < 0.05 at 95% confidence level.

### Results

#### Socio-demographic characteristics of study participants

A total of 244 DM patients participated in the study. However, 29 (11.9%) were unable to provide stool sample. Hence data from 215 participants was complete for analysis. From the total of 215 participants, 56.7% (122/215) were male and 43.3% (93/215) were female. Their mean age was 36.02 years with standard deviation of 9.165 (Table 1).

**Table 1:** Socio-demographic characteristics of diabetic patients in Arba Minch hospital from March to May, 2017 (n = 215)

Characteristics	Frequency (percentage)
Age of respondents	
16–25	28 (13.0%)
26–35	75 (34.9%)
36–45	80 (37.2%)
≥ 46	32 (14.9%)
Sex	
Male	122 (56.7%)
Female	93 (43.3%)
Marital status	
Single	70 (32.6%)
Married	145 (67.4%)
Educational status	
Unable to read and write	42 (19.5%)
Primary	63 (29.3%)
Secondary and above	110 (51.2%)
Occupation	
Government employee	58 (27.0%)
Merchant	56 (26.0%)
Farmer and labor	26 (12.1%)
Student	20 (9.3%)
House wife	55 (25.6%)
Residence	
Urban	181 (84.2%)
Rural	34 (15.8%)

#### Prevalence of intestinal parasites

Among 215 study participants, IPs were detected from 42 (19.5%) diabetic patients and the remaining 173 (80.5%) were negative for IP. Out of the IPs detected, *Cryptosporidium* spp was with the highest frequency (18, 8.4%) followed by *A.lumbricoides* (8, 3.7%) and *G. lamblia* (6, 2.8%) (Table 2).

**Table 2:** Prevalence of intestinal parasites among diabetic patients in Arba Minch hospital from March to May, 2017 (n = 215)

Type of parasite	Frequency (%)
*Cryptosporidium* spp.	18 (8.4)
*Ascaris lumbricoides*	8 (3.7)
*Hook worm*	4 (1.9)
*Trichuris trichuria*	4 (1.9)
*Gardia lamblia*	6 (2.8)
*Teania* spp	2 (0.9)

#### Factors associated with intestinal parasites

In the present study, Male participants showed higher infection rate (27, 12.6%) as compared to female participants (15, 6.9%). However, the difference was not statistically significant (p = 0.273). Participants with age group 36–45 years were more affected than the other group (17, 7.9%) but the difference was not significant. The study showed that intestinal parasitic infections were significantly associated with presence of domestic animals in the house (AOR = 2.857, 95% CI = 1.290–6.330, p = 0.010), manifestation of abdominal pain (AOR = 3.716, 95% CI = 1.632–8.459, p = 0.002) and farmer and labor occupation (AOR = 3.695, 95% CI = 1.082–12.618, p = 0.037) (Table 3).

**Table 3:** Factors associated with intestinal parasitic infection among DM patients in Arba Minch Hospital from March–May, 2017

Variables	Number examined	Rate of IPIs (%)	COR (95%CI)	p-value	AOR (95%CI)	p-value
Age						
16–25	28	2 (0.9)				
26–35	75	14 (6.5)	0.335 (0.071–1.581)	0.167	0.012 (0.001–0.168)	0.061
36–45	80	17 (7.9)	0.285 (0.061–1.323)	0.109	0.357 (0.107–1.188)	0.093
≥ 46	32	9 (4.2)	0.197 (0.038–1.005)	0.051	0.431 (0.144–1.285)	0.131
Sex						
Male	122	27 (12.6)	0.677 (0.336–1.361)	0.273		
Female	93	15 (6.9)				
Marital status						
Single	70	17 (7.9)				
Married	145	25 (11.6)	1.540 (0.768–3.087)	0.224	2.101 (0.987–4.471)	0.054
Educational status						
Un able to read and write	42	11 (5.1)	0.626 (0.270–1.453)	0.276		
Primary	63	11 (5.1)	1.051 (0.467–2.364)	0.905		
Secondary and above	110	20 (9.3)				
Occupation						
Governm’t	58	8 (3.7)	1.389 (0.504–3.825)	0.525		
Merchant	56	11 (5.1)	0.909 (0.351–2.353)	0.844		
Farmer and labor	26	10 (4.7)	0.356 (0.125–1.012)	0.053	3.695 (1.082–12.618)	0.037
Student	20	3 (1.4)	1.259 (0.309–5.136)	0.748		
Housewife	55	10 (4.7)				
Residence						
Urban	181	33 (15.3)				
Rural	34	9 (4.2)	0.619 (0.265–1.449)	0.269		
Food source						
At home	185	36 (16.7)	1.035 (0.394–2.718)	0.945		
From hotel	30	6 (2.8)				
Habit of eating raw vegetables						
Yes	175	34 (15.8)	1.037 (0.439–2.451)	0.934		
No	40	8 (3.7)				
Water source						
Pipe water	211	40 (18.6)				
River/other	4	2 (0.9)	0.234 (0.032–1.711)	0.152	0.173 (0.018–1.645)	0.127
Swimming habit						
Yes	37	7 (3.3)	1.049 (0.426–2.585)	0.917		
No	178	35 (16.3)				
Domestic animal at home						
Yes	80	22 (10.2)	2.181 (1.102–4.317)	0.025	2.857 (1.290–6.330)	0.010
No	135	20 (9.3)				
Washing hands after toilet						
Yes	180	33 (15.3)				
No	35	9 (4.2)	1.542 (0.661–3.596)	0.316		
Abdominal pain						
Yes	62	19 (8.8)	2.497 (1.242–5.022)	0.010	3.716 (1.632–8.459)	0.002
No	153	23 (10.7)				

### Discussion

The magnitude of intestinal parasitosis in the present study (19.5%) was similar with findings from Nigeria (18.7%) [10]. However, it was lower than the prevalence reported in Iran (24.4% to 26.3%) [6, 7] and Egypt (25%) [11]. Difference in laboratory techniques used might bring these magnitude variations; for example, the study conducted in Iran used Kinyoun acid-fast staining to detect Cryptosporidium, and modified trichrome stain (Ryan-Blue) for detection of Microsporidia [6]. The modified Ziehl-Neelson diagnostic technique, we used, couldn’t detect Microsporidia which in turn lowers the overall IP prevalence. The number of specimens collected and examined in order to rule out infection also matters. In the present study only single specimen was collected and examined from each participant despite it is recommended to examine specimens collected in three non-consecutive days [15].

Oocysts of *Cryptosporidium* spp (8.4%) were the most frequently detected parasites in the present study. The prevalence was higher than study findings conducted in Cameroon (0.67%) [9], Iran (2.45%) [7] and Sohag University hospitals, Egypt (5%) [11]. *Giardia lamblia* (3.6%) and *Entamoeba histolytica* (6.7%) were the commonest parasites detected from diabetic patients according to studies from Cameroon and Iran respectively [9, 11]. High geographical distribution of *Cryptosporidium* spp in Ethiopia might bring this difference [16]. Zoonotic transmission of *Cryptosporidium* may also have a role as we find significant association between intestinal parasitosis and presence of domestic animals in the house (p = 0.010). Rate of *Cryptosporidium* infection is associated with level of immune depletion. Hence having immune profile data of study participants would help us to give firm justification about high rate of *Cryptosporidiosis* in the present study.

Male participants were at higher risk of acquiring IP infection (22.1%) compared to females (16.1%) but the difference was not significant (p = 0.273). This goes in line with findings from Iran and Nigeria [6, 7, 11]. Diabetic patients who reported presence of domestic animals in the house were approximately 3 times (AOR = 2.857; 95% CI 1.290–6.330 p = 0.010) at higher risk of acquiring IP infection. The fact that *Cryptosporidium* spp are the most common findings, which are transmitted to humans from cattle, makes the association justifiable. Gastrointestinal discomfort is a common clinical manifestation during IP infection that DM patients who were infected with IP were 3.7 times (AOR = 3.716; 95% CI 1.632–8.459, p = 0.002) at higher risk of having abdominal pain in the present study. DM patients with farmer and labor occupation were also at higher risk of infection (p = 0.037) as most intestinal parasites are transmitted when contacting contaminated soil and water. According to the present study, level of education, occupation, and source of food and water, swimming habit, habit of eating raw fruits/vegetables were not significantly associated with intestinal parasitic infections among diabetic patients. The Small number of participants we recruited is a limitation for not generating strong data about factors associated with intestinal parasitosis.

### Conclusion

In conclusion we, probably for the first time, have shown the rate of intestinal parasitic infections among diabetic patients in Ethiopia. The magnitude of intestinal parasitic infections among DM patients in Arba Minch Hospital was considerable when viewed from the patients’ side. This is because DM causes immune dysfunction which enables parasites to act aggressively and cause severe pathology. Hence, we recommend routine screening and prompt treatment for intestinal parasitosis in order to improve the health of diabetic patients.

## Limitations

Magnitude of intestinal parasitosis among different types of DM, Immunological profile of patients and parasite load were not assessed in the present study, all because of logistic problems.

## Abbreviations

AMH: Arba Minch Hospital
AOR: adjusted odds ratio
COR: crude odds ratio
DM: diabetes mellitus
IP: intestinal parasites
